# Scaling Analysis of Seismic Ground Motion Signals

**DOI:** 10.3390/e28090972

**Published:** 2026-09-01

**Authors:** Giuliana Paradiso, Federica Di Michele, Matteo Colangeli, Bruno Rubino, Lamberto Rondoni

**Affiliations:** 1 Department of Mathematical Sciences, Politecnico di Torino, Corso Duca degli Abruzzi 24, 10129 Turin, Italy; lamberto.rondoni@polito.it; 2 Istituto Nazionale di Geofisica e Vulcanologia, 20133 Milan, Italy; federica.dimichele@ingv.it; 3 Department of Information Engineering, Computer Science and Mathematics, University of L’Aquila, Via Vetoio, 67100 L’Aquila, Italy; matteo.colangeli1@univaq.it (M.C.); bruno.rubino@univaq.it (B.R.); 4 INFN, Sezione di Torino, Via P. Giuria 1, 10125 Turin, Italy

**Keywords:** dissipative phenomena, non-equilibrium statistical mechanics, seismic ground motion, moment scaling, anomalous diffusion, phase detection

## Abstract

Earthquakes exhibit well-documented statistical regularities at the catalogue level, such as the Gutenberg–Richter magnitude–frequency relation and the Omori–Utsu aftershock decay, often interpreted as signatures of seismicity as a driven, dissipative system far from thermodynamic equilibrium. However, whether comparable signatures, such as scale invariance and anomalous diffusion, can be detected directly within individual ground motion recordings remains an open question. This work investigates whether acceleration, velocity, and displacement signals recorded during the 2009 $M_{w}$ 6.1 L’Aquila earthquake display statistical properties consistent with non-equilibrium complex systems, and whether different seismic phases carry distinct, reproducible statistical signatures. P- and S-wave onset times are estimated using AR-AIC, with adaptive search windows centred on theoretical arrivals from the CRUST1.0 velocity model. Coda onset is determined using three complementary criteria combined into a median ensemble, enabling the segmentation of each recording into up to five temporal windows. Displacement moment scaling is analysed for each window and signal type, within the framework of strong anomalous diffusion, yielding the scaling exponents ζ(q). Robustness is systematically assessed against the choice of coda onset method, the empirical thresholds defining coda onset and end, the sub-interval of τ used in the moment scaling fit, and the filter band applied to the ground motion signals. Evidence for anomalous diffusion is nuanced: both its sign and magnitude depend on the seismic phase, with only a subset of configurations remaining stable across all segmentation schemes tested. These results indicate that anomalous scaling signatures, when present, are not universal, and that systematic robustness analyses are essential to distinguish genuine physical effects from segmentation artefacts.

## 1. Introduction

Earthquakes are among the most energetic manifestations of the nonlinear dynamics of the Earth’s crust. Heterogeneous fault systems undergo slow tectonic loading and release accumulated elastic energy through intermittent, abrupt rupture events, a dynamics that is fundamentally chaotic and sensitive to initial conditions [1]. Despite this complexity, statistical regularities emerge at the macroscopic level. Paradigmatic examples include the Gutenberg–Richter frequency–magnitude relation and the Omori–Utsu decay of aftershock rates, which reveal that seismicity exhibits scale-invariant behavior across several orders of magnitude [2,3,4]. These empirical laws suggest that a meaningful characterization of earthquake occurrence requires analyzing the collective dynamics of driven dissipative systems far from the classical realm of thermodynamic equilibria [5,6,7], reflecting an interpretation of the observed scale invariance as a signature of non-equilibrium dynamics rather than an accidental regularity.

In driven dissipative systems far from equilibrium, long-range correlations and stress redistribution across fault networks can produce fluctuations whose statistical properties deviate substantially from Gaussian behavior [8]. Such systems may exhibit heavy-tailed probability distributions, anomalous scaling of statistical moments, and strongly anomalous diffusion, in which the scaling exponent of the *q*-th moment depends nonlinearly on *q* [9,10]. These phenomena are naturally described within the framework of non-equilibrium statistical mechanics and complex systems theory [11,12,13,14,15,16], and have been documented in a variety of physical systems exhibiting multiscale organization and intermittent dynamics [17,18].

Scale invariance and anomalous statistical behavior in seismicity have been extensively characterized at the level of earthquake catalogues, through analyses of magnitude distributions and spatial clustering [19,20]. However, the statistical properties of individual seismic ground motion signals, e.g., acceleration, velocity, and displacement time series recorded by accelerometric networks, have received comparatively less attention from this perspective. A limited number of studies have applied multifractal techniques directly to seismic waveforms or strong ground motion recordings. Padhy [21] employed third-order multifractal detrended fluctuation analysis (MDFA) on seismograms, reporting that multifractal properties are more pronounced when P-, S-, and coda phases are clearly distinguishable within the recording. In a related context, Pnevmatikos et al. [22] applied multifractal wavelet leader analysis to the simulated response of a structure excited by a strong-motion earthquake record, for structural damage detection purposes. These studies, however, rely on detrended fluctuation or wavelet-based multifractal formalisms rather than the moment scaling framework adopted here, and neither systematically segments the signal into distinct seismic phases prior to the scaling analysis, as is done in the present work. Nonetheless, it remains an open question whether the scaling of statistical moments of ground displacement deviates from normal diffusion in ways consistent with out-of-equilibrium dynamics. Whether different seismic phases, namely pre-event noise, primary (P) waves, secondary (S) waves, and scattered coda, carry distinct and reproducible statistical signatures is likewise an open problem, as is the dependence of such signatures on both the signal type and the phase detection methodology.

This work is structured as follows. In Section 2, we describe the large dataset referring to the 2009 L’Aquila earthquake, which serves as our case study. In the same section, we present the methods used to estimate the coda onset time, perform the moment scaling analysis, and compute the scaling exponents. Results are reported in Section 3 and are further analyzed and discussed in Section 4. Conclusions are drawn in Section 5.

## 2. Materials and Methods

The analyzed dataset of three-component ground motion recordings was retrieved from the Italian Accelerometric Archive (ITACA, [23]), the national repository of strong-motion records collected from national, regional, and international seismic networks. ITACA provides uniformly processed three-component time series together with comprehensive metadata including station coordinates, site classification, instrument characteristics, and event parameters.

**Figure 1:**
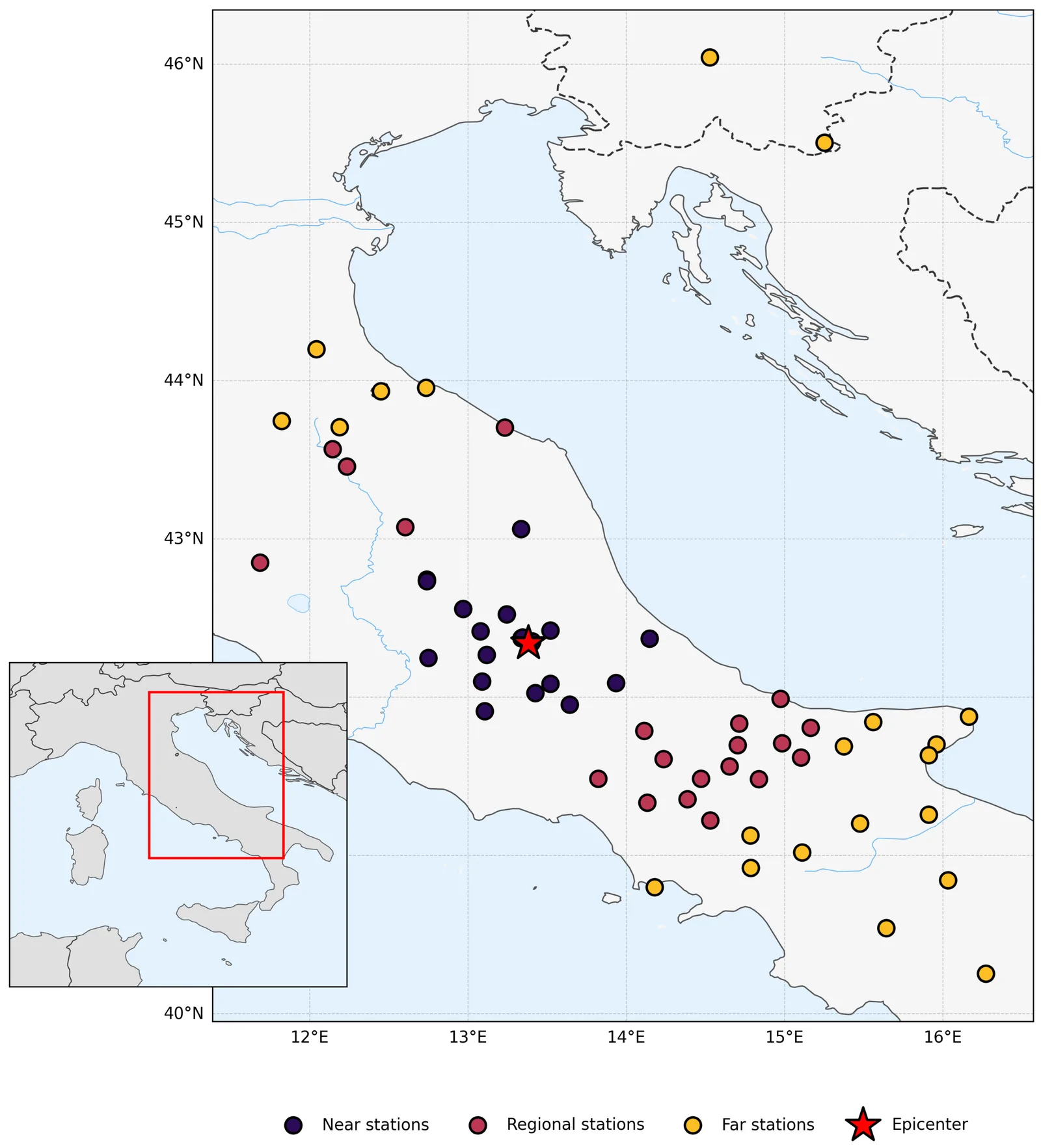
Spatial distribution of the 62 stations used in this study, relative to the 2009 L’Aquila epicenter (red star). Stations are colored by hypocentral-distance group (near, regional, far), following the tertile boundaries used to define the adaptive AR-AIC search windows (Table A1). Inset: location of the study area within Italy.

The dataset corresponds to the L’Aquila earthquake of 6 April 2009 ($M_{w}=6.1$, hypocentral depth 8.3 km, normal faulting). It comprises 192 three-component records from 64 stations belonging to multiple seismic networks, operating at a uniform sampling rate of 200 Hz. Epicentral distances range from approximately 1.8 km to 421 km. A subset of stations employed triggered acquisition, so that the pre-event noise window is absent for those recordings. Two stations were excluded from the analysis: station GSG due to a missing acquisition timestamp, and station TTS due to an anomalous origin time offset incompatible with the segmentation pipeline, leaving 62 stations and 186 three-component records available for analysis. Figure 1 shows the spatial distribution of the 62 stations relative to the epicenter, together with an inset locating the study area within Italy. Stations are colored according to the near/regional/far hypocentral-distance grouping used to define the adaptive AR-AIC search windows (see Section 2.1 and Table A1).

Baseline-corrected acceleration, velocity, and displacement time series were retrieved from the ITACA processing pipeline. A second-order Butterworth bandpass filter had been applied by ITACA prior to archiving, with low-cut frequencies between 0.02 Hz and 0.15 Hz and high-cut frequencies between 20 Hz and 50 Hz depending on station and site conditions. No further amplitude scaling or normalization was applied. For the moment scaling analysis, a baseline correction was applied to each ground motion signal by subtracting its mean value, ensuring zero baseline prior to the computation of displacement increments.

Throughout the pipeline, from phase picking to signal segmentation and moment scaling analysis, all computations are performed in the sample domain to avoid rounding artefacts. Conversion to physical time units (seconds) is applied only at the output stage, for storage and figure axes.

### 2.1. P- and S-Wave Onset Detection

Seismic waves generated by an earthquake propagate through the Earth as two main body wave types. Primary waves (P-waves) are compressional waves in which particle motion is parallel to the propagation direction; they travel fastest and therefore arrive first at recording stations. Secondary waves (S-waves) are shear waves with particle motion perpendicular to the propagation direction; they travel more slowly but carry larger amplitudes and are responsible for most of the damaging ground shaking. The precise identification of P- and S-wave arrival times, known as phase picking, is a prerequisite for segmenting each seismic recording into physically distinct temporal windows for subsequent statistical analysis.

**Figure 2:**
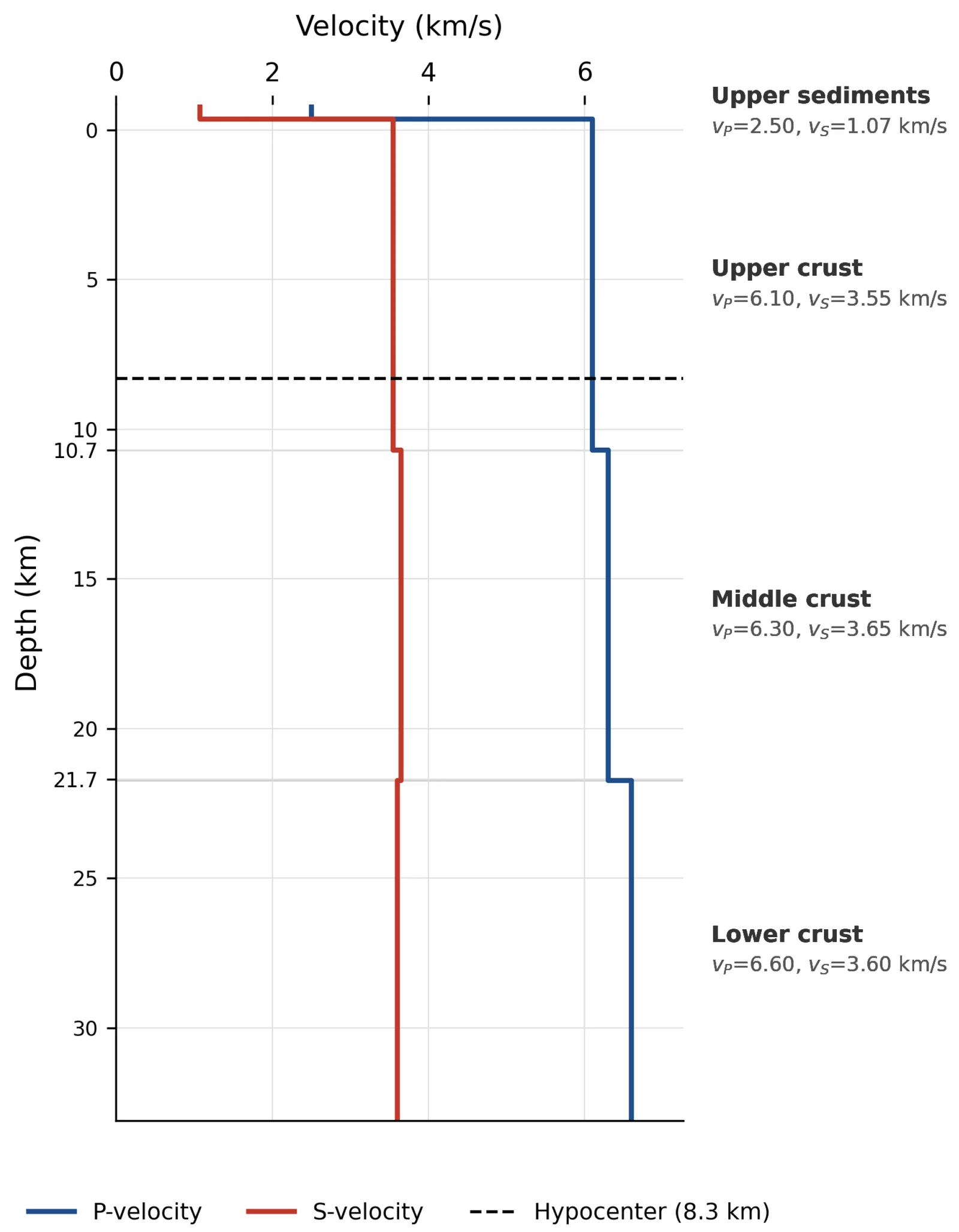
CRUST1.0 P- and S-wave velocity-depth profile at the L’Aquila epicenter. Velocities are shown as step functions reflecting the layered crustal structure; depth values at layer boundaries are marked on the vertical axis. The dashed line indicates the hypocenter depth (8.3 km), separating the layers traversed by the ray path to the surface from those below.

The phase picking and segmentation pipeline is based on the Autoregressive–Akaike Information Criterion (AR-AIC) method, implemented via the ar_pick routine in ObsPy [24]. Theoretical P- and S-wave arrival times are estimated at the station level, independently of signal type, as they depend solely on station location and source parameters, using straight-ray propagation through a layered crustal velocity model extracted from CRUST1.0 [25], illustrated in Figure 2 for the epicenter. Station-specific average velocities are computed as thickness-weighted means over the crustal layers traversed by the ray path from the hypocenter to the surface:

(1) $$v_{P}=\frac{\sum_{k\in T}v_{P}^{\left(k\right)}\;h_{k}}{\sum_{k\in T}h_{k}},\;v_{S}=\frac{\sum_{k\in T}v_{S}^{\left(k\right)}\;h_{k}}{\sum_{k\in T}h_{k}}$$

where T denotes the set of traversed layers and $h_{k}$ is the effective thickness of layer *k*. The theoretical arrival times are then:

(2) $$t_{P}^{\mathrm{theo}}=t_{0}+\frac{d_{\mathrm{hypo}}}{v_{P}},\;t_{S}^{\mathrm{theo}}=t_{0}+\frac{d_{\mathrm{hypo}}}{v_{S}}$$

where $t_{0}$ is the earthquake origin time and $d_{\mathrm{hypo}}=\sqrt{d_{\mathrm{epi}}^{2}+h^{2}}$ is the hypocentral distance, with $d_{\mathrm{epi}}$ the epicentral distance and *h* the hypocenter depth. These theoretical estimates define adaptive search windows within which AR-AIC picking is applied.

Stations are partitioned into three distance bins based on the tertiles of the hypocentral distance distribution, and window half-widths are assigned accordingly: $\Delta t_{P}\in \left\{3,5,7\right\}$ s and $\Delta t_{S}=2\;\Delta t_{P}$ for near, intermediate, and far stations, respectively. The factor of two for S-wave windows reflects the more emergent character of S-wave onsets compared to the typically impulsive P-wave arrivals. A minimum separation gap of 0.5 s is enforced between the end of the P-wave search window and the start of the S-wave search window to prevent phase misidentification.

The ar_pick algorithm is applied with the following fixed parameters, (Table 1) corresponding to those used in the official ObsPy test suite for ar_pick and adopted here as validated default settings rather than tuned specifically to this dataset, consistent with the algorithm’s design, which, as noted in Beyreuther et al. [24], requires comparatively little site-specific tweaking and is intended to be applicable across large, diverse datasets.

Picks with missing values or physically inconsistent ordering ($t_{S}\leq t_{P}$) are discarded prior to signal segmentation.

### 2.2. Signal Segmentation

The subsequent segmentation into temporal windows includes coda onset detection, coda end detection, window definition, and quality control.

#### 2.2.1. Coda Onset Detection

Four independent methods were implemented to estimate the coda onset time $t_{\mathrm{coda}}$, defined as the transition from coherent S-wave energy to diffuse scattered waves. Unlike P- and S-wave arrivals, which correspond to a physically sharp onset, the coda onset marks a gradual transition without an objectively defined ground truth against which a single detection criterion could be validated. Four independent methods, each anchored to a different physical principle, phase timing (Rautian), cumulative energy (Arias), and instantaneous amplitude (envelope decay), are therefore implemented, together with an ensemble estimate (median), to assess the sensitivity of the downstream moment scaling results to this methodological choice, consistent with the robustness framework adopted throughout this work.

The Rautian method [26] defines coda onset from the empirical observation that the coda lapse time—the elapsed time measured from the earthquake origin—is approximately twice the S-wave travel time:

(3) $$t_{\mathrm{coda}}=t_{0}+2\left(t_{S}-t_{0}\right)$$

where $t_{0}$ is the earthquake origin time and $t_{S}$ is the detected S-wave arrival time. This method is computationally simple and distance-independent, but does not account for signal amplitude or local site effects.

The Arias intensity method defines coda onset as the time at which 95% of the total cumulative seismic energy has been released (D5–D95 interval end). The Arias intensity is computed as [27]:

(4) $$\mathrm{AI}\left(t\right)=\frac{\pi }{2g}\int_{0}^{t}x^{2}\left(\tau \right)\;d\tau$$

where x(t) is the ground motion time series and g=9.81 m/s^2^. Coda onset is then defined as [28]:

(5) $$t_{\mathrm{coda}}=\arg\min_{t}\left\{\frac{\mathrm{AI}(t)}{{\mathrm{AI}}_{\mathrm{total}}}\geq 0.95\right\}$$

The envelope decay method identifies coda onset by tracking the decay of the instantaneous amplitude envelope, computed via the Hilbert transform [29]:

(6) $$E\left(t\right)=\sqrt{a{\left(t\right)}^{2}+H{\left[a\left(t\right)\right]}^{2}}$$

where H denotes the Hilbert transform. The envelope is smoothed with a 1-s moving average. Coda onset is defined as the first time the smoothed envelope falls below 30% of the S-wave peak amplitude:

(7) $$t_{\mathrm{coda}}=\arg\min_{t}\left\{E_{\mathrm{smooth}}\left(t\right)<0.3\;E_{\mathrm{peak}},\;t>t_{S}\right\}$$

where α=0.3 is an empirically chosen threshold selected to ensure that the coda onset is identified after the main S-wave energy has decayed substantially, while remaining above the ambient noise level.

The median ensemble method provides a robust estimate by taking the median of the three independent coda onset times:

(8) $$t_{\mathrm{coda}}^{\mathrm{median}}=\mathrm{median}\;\left(t_{\mathrm{coda}}^{\mathrm{Rautian}},\;t_{\mathrm{coda}}^{\mathrm{Arias}},\;t_{\mathrm{coda}}^{\mathrm{Envelope}}\right)$$

The median is preferred over the mean for its robustness to outliers arising from anomalous picks or low signal-to-noise conditions. All coda onset estimates are subject to the physical constraint $t_{S}+1\;s\leq t_{\mathrm{coda}}\leq t_{\mathrm{end}}-1\;s$.

#### 2.2.2. Coda End Detection

Coda end times are estimated using two methods, each paired with its corresponding coda onset method. For the Rautian, envelope, and median onset methods, coda end is identified via envelope decay: the smoothed instantaneous amplitude envelope must fall below 10% of the S-wave peak amplitude and remain below that threshold for a sustained stability duration of 2 s:

(9) $$t_{\mathrm{coda}\_\mathrm{end}}=\arg\min_{t}\left\{E_{\mathrm{smooth}}\left(t\right)<0.10\;E_{\mathrm{peak}}\;\mathrm{for}\;\mathrm{all}\;t^{\prime }\in \left[t,\;t+2\;s\right]\right\}$$

The 10% threshold ensures that the coda end is identified only when the residual seismic energy has decayed to a level substantially below the S-wave peak, consistent with the return to ambient noise conditions. The stability duration of 2 s prevents false detections caused by noise spikes, which could otherwise prematurely terminate the coda window. Both parameters were chosen empirically to balance sensitivity to the signal decay against robustness to noise fluctuations.

For the Arias onset method, coda end is defined as the Arias D99.5 time, the point at which 99.5% of total seismic energy has been released:

(10) $$t_{\mathrm{coda}\_\mathrm{end}}=\arg\min_{t}\left\{\frac{\mathrm{AI}(t)}{{\mathrm{AI}}_{\mathrm{total}}}\geq 0.995\right\}$$

This formulation is internally consistent with the Arias coda onset definition, as both boundaries are derived from the same cumulative energy curve. All coda end estimates are subject to the constraint $t_{\mathrm{coda}}+1\;s\leq t_{\mathrm{coda}\_\mathrm{end}}\leq t_{\mathrm{end}}$.

#### 2.2.3. Definition of Analysis Windows

The detected phase boundaries are used to partition each recording into up to five non-overlapping temporal windows:

(11) $$\begin{array}{cc} W_{\mathrm{pre}} & =\left[\max\left(0,\;t_{P}-5\;s\right),\;t_{P}\right] \end{array}$$

(12) $$\begin{array}{cc} W_{P} & =\left[t_{P},\;t_{S}\right] \end{array}$$

(13) $$\begin{array}{cc} W_{S} & =\left[t_{S},\;t_{\mathrm{coda}}\right] \end{array}$$

(14) $$\begin{array}{cc} W_{\mathrm{coda}} & =\left[t_{\mathrm{coda}},\;t_{\mathrm{coda}\_\mathrm{end}}\right] \end{array}$$

(15) $$\begin{array}{cc} W_{\mathrm{post}} & =\left[t_{\mathrm{coda}\_\mathrm{end}},\;t_{\mathrm{end}}\right] \end{array}$$

The post-event window $W_{\mathrm{post}}$ is created only when a valid $t_{\mathrm{coda}\_\mathrm{end}}$ is detected before the end of the recording; otherwise the coda window extends to $t_{\mathrm{end}}$. For triggered recordings in which acquisition began after the P-wave or S-wave arrival, the available windows are restricted accordingly: if $t_{P}^{\mathrm{theo}}<0$, the pre-event and P-wave windows are not created; if additionally $t_{S}^{\mathrm{theo}}<0$, only the coda and post-event windows are available. Any window whose duration falls below 2 s is discarded, as excessively short windows would restrict the maximum time lag $\tau_{\max}$ available for the moment scaling analysis, which is bounded by the shortest window duration in the ensemble.

#### 2.2.4. Quality Control

All automatically detected picks are subjected to three quality control criteria. First, a monotonicity check verifies that P- and S-wave arrival times increase monotonically with hypocentral distance across stations, as expected from finite wave propagation velocity. Second, a minimum signal-to-noise ratio of SNR≥3 is required for each component, computed as the ratio of RMS amplitude over the first 5 s following phase onset to the RMS amplitude of the pre-event window. Third, a peak ground motion timing check verifies that the peak absolute amplitude of each component, retrieved from the ITACA metadata, falls within the S-wave window $\left[t_{S},t_{\mathrm{coda}}\right)$, where seismic energy is physically expected to be highest. Components failing one or more criteria are flagged for visual inspection.

### 2.3. Moment Scaling Analysis

The scaling behaviour of statistical moments is investigated to detect signatures of anomalous or strongly anomalous diffusion, following the framework of Vollmer et al. [9]. For a stochastic process x(t) representing the seismic signal (acceleration, velocity, or displacement), the *q*-th order moment of the increments $\Delta x\left(\tau \right)=x\left(t_{0}+\tau \right)-x\left(t_{0}\right)$ is expected to scale as:

(16) $$M_{q}\left(\tau \right)={\langle |\Delta x\left(\tau \right)|}^{q}\rangle \;∼\tau^{\zeta (q)},$$

where τ is the time lag and 〈·〉 denotes ensemble averaging. For normal diffusion, ζ(q)=q/2 for all *q*, yielding a linear scaling spectrum. Deviations from this reference indicate anomalous diffusion, while strong anomalous diffusion is characterised by a piecewise-linear spectrum with a crossover at a critical moment order α [9]:

(17) $$\zeta \left(q\right)=\left\{\begin{array}{cc} \xi \;q & q\leq \alpha ,\\ \zeta_{0}\;q-\alpha \left(\zeta_{0}-\xi \right) & q>\alpha , \end{array}\right.$$

where $\xi <\zeta_{0}$. The lower branch is governed by the bulk of the displacement distribution, while the upper branch is dominated by rare large fluctuations in the tails. The analysis is performed independently for each signal type (acceleration, velocity, displacement), each temporal window (pre-event, P-wave, S-wave, coda, and post-event when present) and each coda onset definition (Rautian, Arias, envelope decay, and median ensemble), enabling a systematic comparison of scaling behaviour across dynamical regimes and methodological choices.

#### 2.3.1. Spatial Ensemble Averaging

Estimates of moment scaling exponents are obtained by spatial ensemble averaging across the recording network. For a given temporal window, the ensemble-averaged moment of order *q* at time lag τ is:

(18) $$\langle M_{q}\left(\tau \right)\rangle =\frac{1}{N}\sum_{i=1}^{N}{\left|\Delta x_{i}\left(\tau \right)\right|}^{q},$$

where *N* is the number of station-component pairs contributing to the window, and $\Delta x_{i}\left(\tau \right)=x_{i}\left(t_{0,i}+\tau \right)-x_{i}\left(t_{0,i}\right)$ is the increment of the *i*-th ground motion time series, with $t_{0,i}$ denoting the window start time of the *i*-th trace. The time lag τ spans a common range for all traces within a given window, with $\tau_{\max}$ set independently for each of the five temporal windows as the shortest trace duration in the corresponding ensemble. The three orthogonal components recorded at each station are treated as independent ensemble members, as each component samples a different projection of the wavefield and therefore provides an independent realisation of the underlying process.

#### 2.3.2. Computation of Moments and Scaling Exponents

Moments are computed for moment orders q∈[0.25,10] with step Δq=0.25, yielding 40 values. Time lags τ are distributed logarithmically between $\tau_{\min}=0.5$ s and $\tau_{\max}=\min_{i}\left({\mathrm{duration}}_{i}\right)$, with approximately 20 points per decade in log scale and a minimum of 30 points total.

The scaling exponent ζ(q) is estimated by ordinary least squares regression on the log-transformed data:

(19) $$\log_{10}\langle M_{q}\left(\tau \right)\rangle =\zeta \left(q\right)\;\log_{10}\tau +c,$$

excluding points where $M_{q}\left(\tau \right)<10^{-15}$ or where $M_{q}\left(\tau \right)$ is undefined. If fewer than 3 valid points remain for a given *q*, the corresponding exponent is set to undefined. The goodness of fit is assessed via the coefficient of determination $R^{2}$, which serves as a quality metric for the estimated scaling spectrum ζ(q). The standard error of the regression slope provides an uncertainty estimate for ζ(q), reported as error bars in the corresponding figures.

### 2.4. Sensitivity Analysis

To assess the extent to which the moment scaling results depend on methodological choices made throughout the segmentation and analysis pipeline, three empirical parameters are varied one at a time relative to their baseline value: (i) the coda onset and coda end amplitude thresholds; (ii) the sub-interval of τ used in the log-log regression for the P-wave window, where the intrinsically narrow dynamic range constrains the reliability of the fit; and (iii) the band-pass filter frequencies applied to the ground motion signals prior to analysis. For each alternative configuration, the scaling exponents ζ(q) are re-estimated and compared against the corresponding baseline value using a pointwise *z*-score,

(20) $$z\left(q\right)=\frac{\left|\zeta (q;\;\mathrm{alt})-\zeta (q;\;\mathrm{base})\right|}{\sqrt{\sigma_{\mathrm{alt}}^{2}\left(q\right)+\sigma_{\mathrm{base}}^{2}\left(q\right)}},$$

where σ denotes the standard error of the corresponding fit. This metric expresses the deviation between configurations in units of the combined estimation uncertainty, rather than as an absolute or relative difference, so that a given numerical deviation is judged relative to how precisely each exponent is constrained.

#### 2.4.1. Coda Threshold Sensitivity

The coda onset envelope threshold (baseline 30%, Equation (7)) and the coda end amplitude threshold (baseline 10%, Equation (9)) are each varied independently, holding the other fixed at its baseline value. The envelope threshold, which affects coda onset for the Envelope and Median methods only (Rautian and Arias are unaffected by construction), is tested at 20%, 25%, 35%, and 40%. The coda end threshold, which affects the Rautian, Envelope, and Median methods (Arias uses an independent energy-based end criterion, Equation (10)), is tested at 5%, 15%, and 20%. For each alternative threshold, the affected coda methods are re-segmented and the moment scaling analysis is repeated for the S-wave and coda windows, the only windows whose boundaries depend on these thresholds.

#### 2.4.2. P-Wave τ Sub-Interval Sensitivity

Since the P-wave window is intrinsically short ($\tau_{\max}\approx 2.1$–2.4 s), the log-log regression used to estimate ζ(q) spans less than one decade of τ. To assess whether a single power-law adequately describes the moment scaling over this narrow range, the available τ values are split at their midpoint in $\log_{10}\tau$ space into a lower and an upper sub-interval, and ζ(q) is re-estimated independently on each half using the same regression procedure as the baseline fit (Equation (19)). Since halving the sample size inflates the standard error of each sub-interval fit, the *z*-score defined in Equation (20) alone provides limited statistical power to detect a systematic deviation between sub-intervals, particularly at high *q*. A complementary test is therefore used to detect a directional trend that would not be captured by only comparing individual *z*-scores: a two-sided binomial sign test on the direction of the difference between the upper- and lower-interval estimates, ζ(q;upper)−ζ(q;lower), across all analysed values of *q*. Under the null hypothesis that this sign is equally likely to be positive or negative by chance, a consistent sign across many values of *q* is unlikely to arise from fit-to-fit noise alone, and instead indicates a systematic departure from a single power-law scaling within the P-wave window.

#### 2.4.3. Filter Band Sensitivity

The waveforms used throughout this work are the pre-filtered acceleration time series archived by ITACA, obtained by applying a second-order Butterworth bandpass filter prior to archiving, with per-station low-cut and high-cut corner frequencies in the ranges 0.02–0.15 Hz and 20–50 Hz, respectively.

To assess the sensitivity of the moment scaling results to this choice, three filter band configurations are tested, all applied to the non-filtered waveforms rather than to the pre-filtered archived signals used in the baseline analysis: (i) a near full-band configuration, with a low-cut corner close to 0 Hz and a high-cut corner just below the Nyquist frequency, approximating the absence of filtering within the constraint that ar_pick requires strictly positive, finite corner frequencies; (ii) a narrow band, with fixed corners at 0.15 Hz and 20 Hz; and (iii) a wide band, with fixed corners at 0.02 Hz and 50 Hz. The narrow and wide bands are anchored to the extremes of the per-station range already employed by ITACA.

For each configuration, the chosen corner frequencies are applied twice: first directly to the signal amplitudes, and again internally by ar_pick during phase picking, consistent with how the baseline corner frequencies are used. The resulting moment scaling exponents are compared against the baseline over the P-wave, S-wave, and coda windows.

## 3. Results

The results are organized in two parts. Section 3.1 reports the phase detection and signal segmentation outcomes, including residual statistics and quality control results. Section 3.2 presents the moment scaling spectra ζ(q) estimated across seismic phases and signal types.

### 3.1. Phase Detection and Signal Segmentation

Phase detection was performed using AR-AIC across 62 stations and the three signal types, as described in Section 2.1. Complete detection, residual, and quality control statistics are reported in Appendix A.

P-wave residuals are comparable across signal types, with standard deviations ranging from 1.92 s to 2.32 s. The mean P-wave offset is positive for acceleration (+0.55 s) and velocity (+0.23 s), and negative for displacement (−0.61 s). S-wave residuals show larger dispersion, with displacement exhibiting the highest standard deviation (7.04 s, compared to 5.36 s and 5.83 s for acceleration and velocity). Residual statistics for all signal types are reported in Table A2.

Quality control results are reported in Table A3. Peak check violations range from 6.7% to 48.2% across signal types and coda methods, with the envelope method showing systematically higher violation rates. MonoP and MonoS check violations are high across all signal types (82.3–87.1% and 85.5–90.3%, respectively). SNR P check violations are negligible for acceleration (0.0%) but increase for velocity (6.7%) and displacement (26.5%). SNR S check violations remain low for acceleration and velocity (0.0%) and reach 2.5–2.6% for displacement.

**Figure 3:**
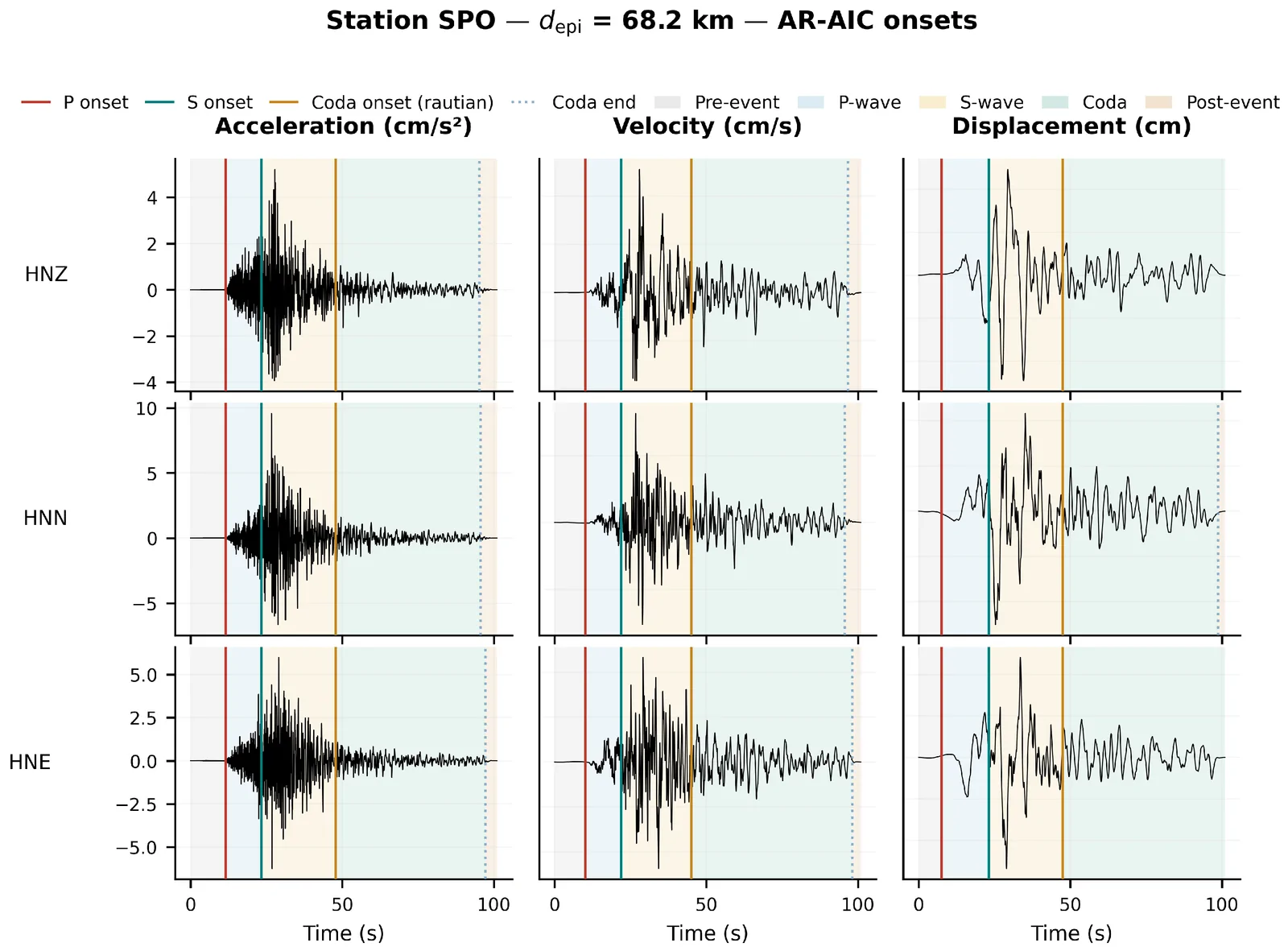
Signal segmentation for station SPO ($d_{\mathrm{epi}}=68.2$ km), using AR-AIC onset detection and Rautian coda onset method, for acceleration, velocity, and displacement signals. Vertical lines indicate the detected P-wave onset (red), S-wave onset (teal), coda onset (orange), and coda end (blue dashed). Colored backgrounds denote the pre-event, P-wave, S-wave, coda, and post-event windows.

To illustrate the segmentation pipeline described above, Figure 3 shows the detected phase onsets and resulting temporal windows for station SPO. This station was selected for illustrative purposes since all five temporal windows are available for this record, whereas triggered-acquisition stations lack the pre-event window.

### 3.2. Moment Scaling and Anomalous Diffusion

Moment scaling spectra ζ(q) were estimated for three temporal windows (P-wave, S-wave, and coda) across three signal types (acceleration, velocity, and displacement). Results are presented for the Rautian coda onset method as the primary reference; the robustness of the scaling exponents with respect to the choice of coda onset definition is assessed by comparison with the Arias, Envelope, and Median methods.

**Figure 4:**
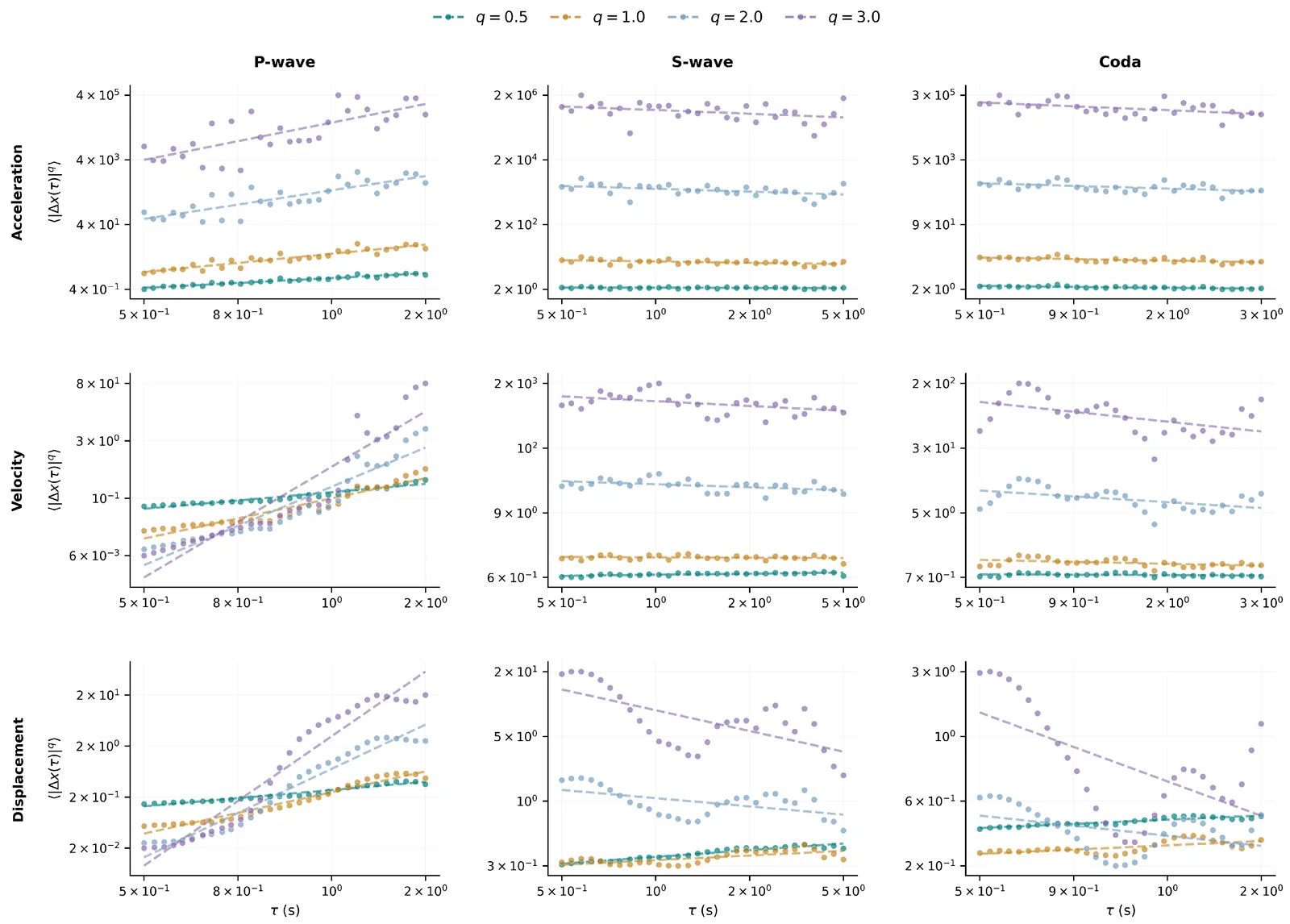
Ensemble-averaged moment scaling curves for the IT-2009-0009 dataset, Rautian coda onset.

**Figure 5:**
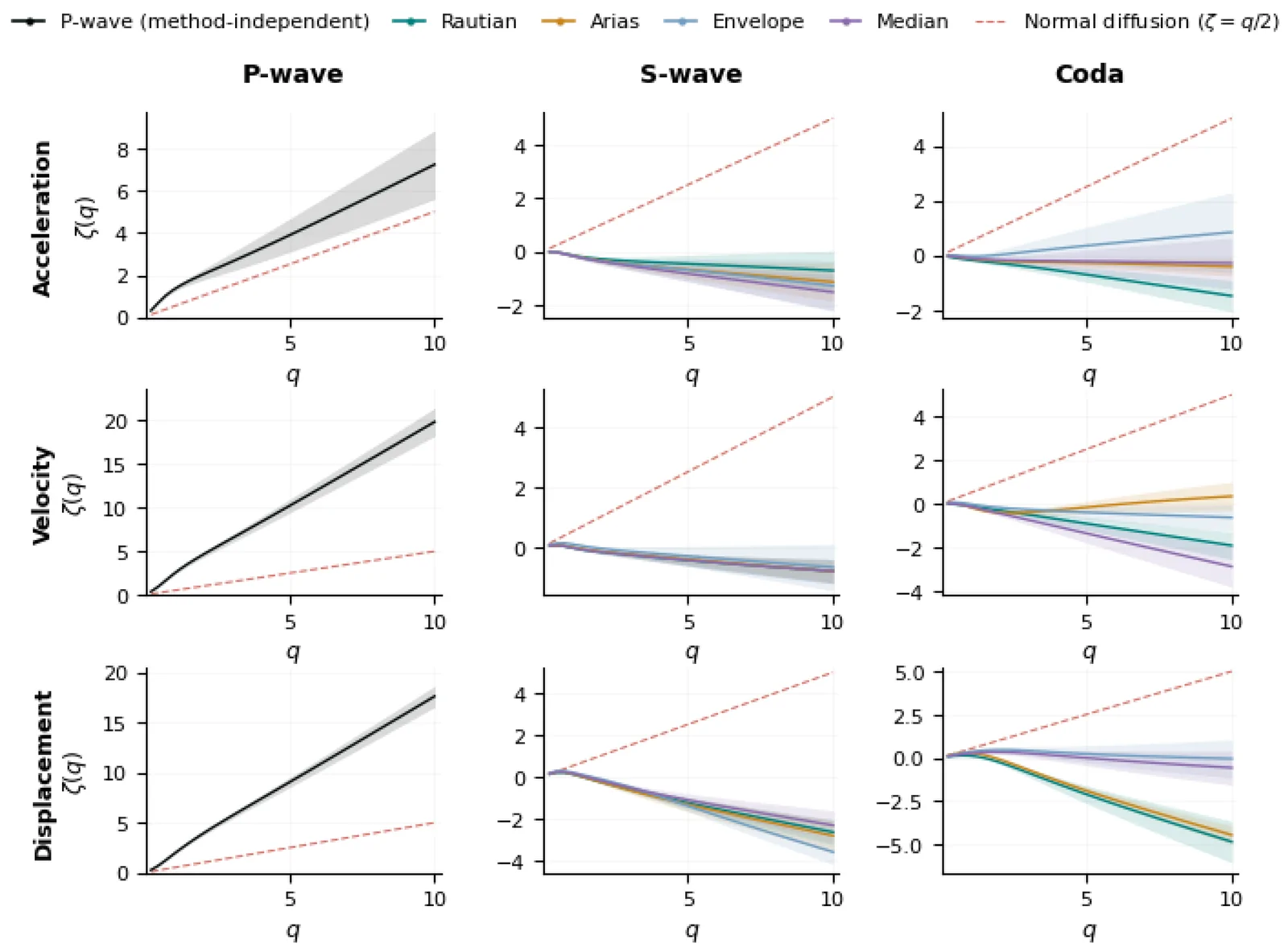
Scaling exponent spectra ζ(q) for the IT-2009-0009 dataset, comparing all four coda onset methods (Rautian, Arias, Envelope, Median). The P-wave column shows a single curve, since P-wave window boundaries do not depend on the coda onset method and results are therefore identical across methods. Shaded bands represent the estimated uncertainty (ζ(q)±σ).

Figure 4 shows the ensemble-averaged moment scaling curves for the AR-AIC segmentation with Rautian coda onset, and Figure 5 the corresponding scaling exponent spectra, comparing all four coda onset methods. Full numerical results for all four coda onset methods are reported in Table 2, Table 3 and Table 4.

In the **P-wave window**, ζ(q) grows monotonically and approximately linearly with *q* for all three signal types, well above the normal diffusion reference ζ(q)=q/2. Acceleration yields ζ(1)=1.28 ($R^{2}=0.87$) and ζ(2)=2.00 ($R^{2}=0.70$), velocity ζ(1)=2.34 ($R^{2}=0.91$) and ζ(2)=4.55 ($R^{2}=0.89$), and displacement ζ(1)=1.80 ($R^{2}=0.96$) and ζ(2)=3.85 ($R^{2}=0.95$). The power-law fits are well-constrained across all signal types. The P-wave window results are identical across all four coda onset methods, as expected given that the P-wave window boundaries are independent of the coda onset definition.

In the **S-wave window**, the scaling structure changes markedly across signal types. Acceleration shows ζ(q) near zero and decreasing with *q*, with ζ(1) ranging from −0.15 to −0.12 and ζ(2) from −0.36 to −0.27 across coda onset methods ($R^{2}=0.19$–0.38). Velocity shows a similar pattern, with ζ(1) close to zero (−0.02 to 0.10) and more negative ζ(2) (−0.05 to −0.17, $R^{2}<0.23$). Displacement yields weakly positive ζ(1) (0.14–0.22, $R^{2}=0.47$–0.61) but negative ζ(2) (−0.11 to −0.25), indicating a non-linear dependence of the exponent on *q* for all three signal types. Results are broadly consistent across the four coda onset methods.

In the **coda window**, acceleration and velocity show negative scaling exponents across all coda onset methods, with ζ(1)∈[−0.17,−0.03] for acceleration and ζ(1)∈[−0.15,−0.01] for velocity. Displacement shows a qualitatively different and method-dependent pattern: ζ(1) is positive (0.11–0.34) under all methods, but ζ(2) is negative under Rautian and Arias and positive under Envelope and Median, indicating sensitivity to the choice of coda onset definition.

The slopes of the acceleration curves themselves carry physical significance: ζ(q) grows markedly with *q* in the P-wave window, remains close to zero in the S-wave window, and stays modest and weakly structured in the coda window, a pattern related to the physical characteristics of each seismic phase in Section 4.2. Figure 4 reveals two additional features that are informative of the underlying dynamics. First, in the P-wave window, the curves for velocity and displacement at different *q* cross one another within the range of τ shown, with the ordering of the curves at short τ reversed by $\tau_{\max}$; this crossover is absent for acceleration, where the curves remain parallel throughout. This behaviour follows directly from the definition of the moment, $M_{q}\left(\tau \right)={\langle |\Delta x\left(\tau \right)|}^{q}\rangle$: raising an increment smaller than unity to a higher power *q* further reduces its value, so lower-*q* curves lie above higher-*q* curves until the increment exceeds unity, at which point the ordering reverses. Velocity and displacement, obtained by integrating acceleration, have smaller increments, which cross this threshold within the P-wave window, whereas those of acceleration remain above it throughout. Second, in the S-wave and coda windows, several curves, most visibly at high *q*, are non-monotonic, exhibiting an internal maximum or minimum rather than following a single power law across the full τ range; at the level of the moment curves, no comparable feature is visible in the P-wave window. This non-monotonicity indicates a visible departure from a strict power law within these windows, offering a possible physical origin for the low $R^{2}$ values already reported for the S-wave and coda fits (Section 4.2): the S-wave and coda windows encompass a superposition of sub-phases, for which a single power-law exponent captures only the leading-order trend. A comparable departure from strict power-law scaling is found for the P-wave window as well, although there it is not visible directly in the moment curves and instead emerges only from the τ sub-interval sensitivity analysis (Section 3.3.2), where it manifests as a systematic dependence of ζ(q) on which portion of the available τ range is used in the fit.

The spectra ζ(q) shown in Figure 5 further clarify the physical picture outlined above. In the P-wave window, ζ(q) increases monotonically and without saturation over the full range of *q* tested, consistent with the increasing dominance of rare, large fluctuations at high moment order discussed in Section 4.2.1. In the S-wave and coda windows, by contrast, ζ(q) starts close to zero at low *q* and decreases, becoming negative at high *q*, with the spread across coda onset methods widening as *q* increases. This decreasing trend is a consequence of the non-monotonic behaviour of the underlying moment curves $M_{q}\left(\tau \right)$ in these windows, discussed above: since a single log-log fit is applied across the full τ range, and higher-order moments weight the declining portion of a non-monotonic curve more heavily, the fitted slope becomes increasingly negative at high *q*. This negative trend should be interpreted as a signature, within a single power-law fit, of the same superposition of sub-phases responsible for the non-monotonicity of $M_{q}\left(\tau \right)$ in these windows.

### 3.3. Sensitivity Analysis

This section reports the outcome of the three sensitivity tests described in Section 2.4: coda threshold sensitivity, P-wave τ sub-interval sensitivity, and filter band sensitivity. Coda threshold tests have been conducted on the acceleration records only. Filter band tests are similarly restricted to acceleration, since the unprocessed ITACA waveforms required for this analysis (Section 2.4.3) are available for acceleration but not for velocity or displacement.

#### 3.3.1. Coda Threshold Sensitivity

Table 5 and Table 6 report the z-scores for the coda onset envelope threshold and the coda end threshold, respectively.

Across both thresholds, z-scores remain below the conventional significance level of z≈2 for all but one configuration ($z_{\max}=2.06$ for the Envelope method under the 20% onset threshold, in the coda window), with the Envelope method consistently the most sensitive of the three affected methods, particularly in the coda window.

The coda end threshold shows an asymmetric effect: reducing the coda window (15%) produces larger z-scores than enlarging it (5%), despite both directions being equidistant from the baseline, consistent with a stricter threshold removing a non-negligible fraction of an already short window while a more permissive one only admits marginal, already-decaying samples.

#### 3.3.2. P-Wave τ Sub-Interval Sensitivity

**Figure 6:**
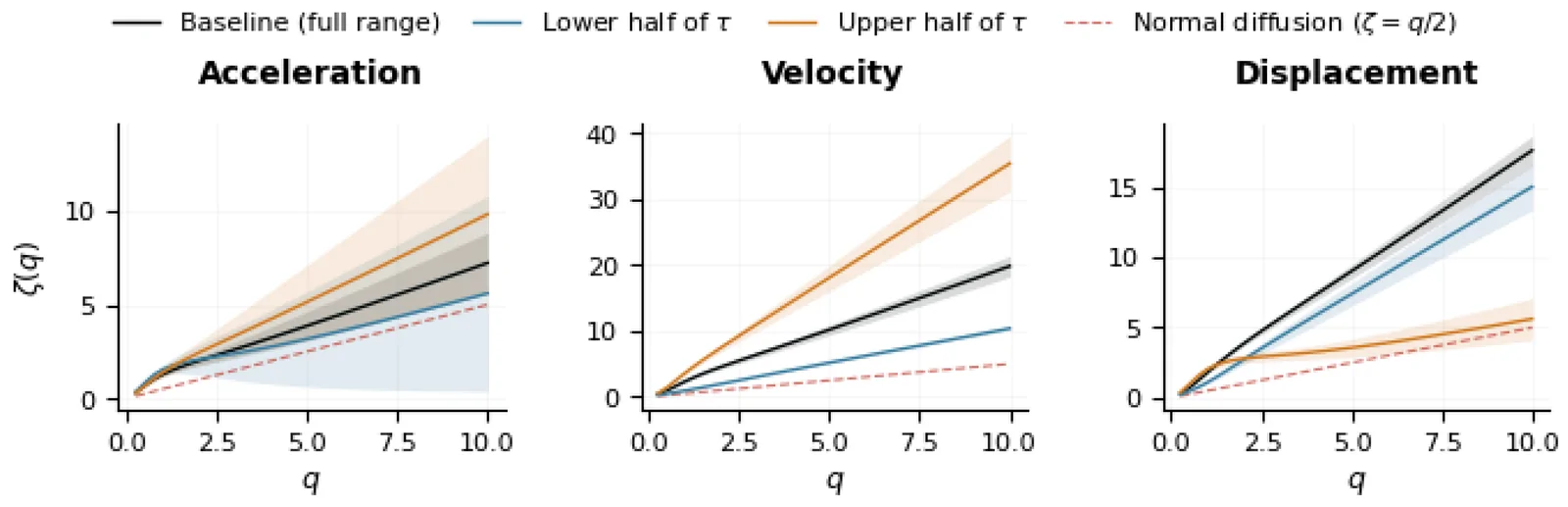
Scaling exponents ζ(q) for the P-wave window, estimated on the full τ range (baseline), the lower sub-interval, and the upper sub-interval of τ, for acceleration, velocity, and displacement. Shaded bands represent the estimated uncertainty (ζ(q)±σ).

Table 7 reports the outcome of the sign test described in Section 2.4.2, and Figure 6 shows ζ(q) estimated on the full τ range, the lower sub-interval, and the upper sub-interval, for acceleration, velocity, and displacement.

For all three signal types, the sign test rejects the null hypothesis of a random, non-systematic difference between the two sub-intervals ($p<10^{-4}$ in all cases), indicating that the moment scaling within the P-wave window is not adequately described by a single power law over its full τ range. The magnitude and character of this departure, however, differ across signal types. For acceleration, ζ(q;upper) exceeds ζ(q;lower) for 35 of 40 tested values of *q*, but the pointwise *z*-score (Equation (20)) remains below 2 throughout, reflecting the combination of systematic trend and inflated uncertainty in each half-sample fit. For velocity, the same directional trend holds for all 40 values of *q*, with *z*-scores reaching approximately 9 at low *q* and remaining above 3.4 across the full range. The P-wave scaling exponents for velocity are the least stable of the three signal types with respect to the choice of τ sub-interval. For displacement, the direction of the deviation reverses at q≈1.25, where ζ(q;lower) and ζ(q;upper) are approximately equal (*z*-score ≈0.01): below this crossover, ζ(q;upper) exceeds ζ(q;lower), while above it the ordering reverses and the deviation grows to *z*-scores of approximately 6–7 at high *q*.

These results indicate that the large P-wave scaling exponents reported at high *q* (Section 4.2.1) should not be interpreted as a stable, well-constrained physical scaling regime, particularly for velocity, where the instability is most pronounced, and for displacement, where the qualitative form of the scaling behaviour itself depends on which portion of the available τ range is considered.

#### 3.3.3. Filter Band Sensitivity

Table 8, Table 9 and Table 10 report the z-scores for the three alternative filter band configurations, for the P-wave, S-wave, and coda windows respectively. The sensitivity to the filter band is uneven across windows. In the P-wave window z-scores exceed the conventional significance level of z≈2 for all three alternative configurations, with $z_{\max}$ reaching 4.88 for the near full-band configuration, 5.24 for the narrow band, and 2.73 for the wide band, the least extreme of the three. In both the S-wave and coda windows, by contrast, z-scores remain modest: all configurations and coda methods stay below z≈1.2 in the S-wave window, and below z≈2 in the coda window with a single exception, the Rautian method under the narrow band configuration ($z_{\max}=2.36$).

This pattern indicates that the moment scaling exponents estimated in the P-wave window are the most sensitive to the choice of filter band, consistent with the sensitivity already observed for this window with respect to the τ sub-interval used in the fit (Section 3.3.2). The S-wave and coda windows, in contrast, remain robust to this methodological choice.

## 4. Discussion

The following sections discuss the phase detection results and the moment scaling findings, interpreting the patterns observed in Section 3 in terms of methodological choices, signal characteristics, and physical implications.

### 4.1. AR-AIC Picking

The most striking feature of the AR-AIC quality control results (Section 3.1) is the prevalence of monotonicity failures: the majority of components violate the expected ordering of arrival times with hypocentral distance (82.3–90.3%). Rather than indicating picking errors, this pattern is more plausibly attributable to the MonoP/MonoS check itself, which assumes a strictly monotonic relationship between arrival time and distance under a simplified, laterally homogeneous velocity structure. Real crustal heterogeneity and path effects, well documented in local earthquake studies through the systematic station corrections required to reconcile observed arrival times with simplified velocity models [30], together with focal mechanism radiation patterns, can all produce non-monotonic arrival sequences that are physically legitimate rather than picking artefacts. The wide and heterogeneous range of epicentral distances spanned by the network (1.80–421.40 km) plausibly increases the influence of path-dependent velocity heterogeneity on the validity of the monotonicity assumption.

A second pattern emerges for displacement signals, which fail the SNR P check more frequently than acceleration or velocity (26.5%), and exhibit the largest S-wave residual dispersion among the three signal types. This is consistent with the integration-induced degradation of high-frequency timing information discussed throughout this work, indicating that doubly-integrated signals systematically carry less reliable timing information than the underlying acceleration record.

The elevated Peak check violation rate observed under the Envelope coda onset method relative to Rautian, Arias, and Median (up to 48.2%, against a maximum of 23.3% for the other three methods) suggests that, for this dataset, the S-wave window boundaries implied by the Envelope method are less consistent with the expected location of the peak ground-motion value than those implied by the other coda definitions; the implications of this discrepancy for the moment scaling results are examined further in Section 4.2.3.

Finally, it is worth acknowledging an interpretive limitation that applies to all AR-AIC residuals discussed in this section: since residuals are computed relative to theoretical arrival times derived from the CRUST1.0 crustal model, an elevated residual may reflect either a genuine picking error or an inaccuracy in the assumed velocity structure, and the two contributions cannot be disentangled with the available data. The comparisons drawn above should therefore be understood as comparisons of picking consistency, not as an absolute validation of AR-AIC against a perfectly known ground truth.

### 4.2. Evidence for Anomalous Scaling

This section addresses whether the moment scaling results presented in Section 3.2 provide evidence for anomalous diffusion, and how this evidence varies across seismic phases, segmentation configurations, and coda onset definitions.

#### 4.2.1. P-Wave Window

Segmentation yields positive scaling exponents for all three signal types in the P-wave window. Acceleration and displacement lie well above the normal diffusion reference, with consistently well-constrained log–log fits ($R^{2}$ up to 0.99). Velocity behaves similarly to acceleration and displacement, with ζ(q) lying above the normal diffusion reference throughout the range of *q* considered.

A caveat applies to the magnitude of the exponents reported across all configurations. The P-wave window is intrinsically short ($\tau_{\max}$ around 2.1–2.4 s), limiting the dynamic range of τ available for the fit. This constrains the reliability of the extrapolated exponents at high moment order: the values reported here, reaching ζ(5)≈9–10 for velocity and displacement, appear large for a physical scaling exponent even where the fit at q=1,2 is well constrained. This caveat is confirmed by the two sensitivity analyses targeting this window (Section 3.3.2 and Section 3.3.3). Although conducted independently and varying unrelated aspects of the pipeline, both point to the same underlying limitation: with only a few seconds of signal and a correspondingly narrow range of τ available for the fit, the estimated exponent is governed by a small number of samples and is easily perturbed by choices that alter which portion of that short window, or which frequency content within it, dominates the regression. The convergence of two unrelated tests on the same conclusion strengthens the case that this fragility is a structural property of the P-wave window itself, rather than an artefact of either sensitivity test individually. Unlike the S-wave and coda windows, therefore, the P-wave scaling exponents reported here should be read as evidence of a departure from normal diffusion, rather than as precisely constrained physical quantities.

The growth of ζ(q) with *q* observed for acceleration in this window is compatible with the impulsive character of the P-wave arrival: a short, high-amplitude transient standing out sharply against the background level within a window lasting only a few seconds. Within the strong anomalous diffusion framework, ζ(q) increases with *q* precisely when high-order moments are dominated by a small number of rare, large fluctuations rather than by the typical behaviour of the signal. Notably, this is plausibly the same signal feature that enables automatic phase picking in this window: AR-AIC identifies the P-wave onset by detecting an abrupt change in the local statistical properties of the signal, with the AIC minimum corresponding to the point where the autoregressive properties change sharply. The growth of ζ(q) with *q* and the effectiveness of AR-AIC picking are therefore compatible with a common physical origin.

#### 4.2.2. S-Wave Window

In the S-wave window, ζ(q) is typically negative and modestly decreasing for acceleration and displacement, but the fits are poorly constrained ($R^{2}$ only modestly above 0.10 in most cases).

The choice of coda onset method, which determines the upper boundary of the S-wave window, has negligible influence on the S-wave scaling results: the four methods yield essentially identical ζ(q) spectra for all three signal types, despite the fact that $\tau_{\max}$ differs across methods as a consequence of their different coda onset estimates. This robustness extends to the filter band applied to the ground motion signal: the S-wave sensitivity analysis (Section 3.3.3) yields z-scores well below the significance threshold across all coda onset methods and all three alternative filter configurations tested, the lowest of the three windows examined.

From a physical standpoint, the S-wave window is the most energetic phase of the recording, with PGA, PGV, and PGD typically reaching their peak values here. Unlike the P-wave window, however, it is not dominated by a single isolated transient: it comprises the direct shear arrival, often superimposed with converted phases and shallow crustal reverberations. This superposition of multiple arrivals is compatible with behaviour closer to, although not coincident with, normal diffusion, and the signal behaves in a more diffuse manner in the statistical sense.

#### 4.2.3. Coda Window

The coda window yields comparatively modest scaling exponents in most configurations, with ζ(1) and ζ(2) typically between approximately −0.4 and +0.4, although departures from this range are frequent and can be substantially larger, with ζ(q) at q=5 reaching values as negative as approximately −2 under certain coda onset method combinations (see the rightmost column in Figure 5). Even accounting for these exceptions, the coda window remains substantially less extreme overall than the P-wave window, where ζ(5) reaches approximately 9–10 for several signal types.

Physically, the coda consists of scattered energy generated by crustal heterogeneities, which progressively decays towards the ambient noise level. As the signal moves further away from the S-wave arrival, it loses the impulsive or energetic structure of the preceding phases and increasingly resembles a stochastic fluctuation around zero, consistent with ζ(q) values that do not depart markedly from the behaviour expected of a process approaching background noise.

One combination stands out as a candidate for a genuine, rather than artefactual, signal: displacement under the Arias coda onset method yields a notably well-constrained fit, with $R^{2}=0.93$ for ζ(1). This is the best-constrained result observed in the coda window; notably, it is also one of the configurations responsible for the more extreme departures from the modest-exponent range noted above, with ζ(q) reaching approximately −2 at q=5.

The clearest pattern concerning the choice of coda onset method is the divergence in Peak check performance already noted in Section 4.1: Envelope is systematically the worst-performing method, while Arias is systematically the best. These patterns are broadly consistent with the conceptual characterisation of the three independent coda onset methods: Rautian is a deterministic, phase-anchored formula, comparatively insensitive to the specific shape of the waveform; Arias is an energy-based criterion anchored to the signal itself; and Envelope is an amplitude-based criterion with only partial phase anchoring. Arias does not perform uniformly well across all configurations, but it yields the best-constrained result in the coda window for displacement. Median, finally, behaves as expected of a robust ensemble combination, with values remaining close to the central tendency of the other three methods throughout.

## 5. Conclusions

This work investigated whether seismic ground motion signals, acceleration, velocity, and displacement, recorded during the 2009 $M_{w}$ 6.1 L’Aquila earthquake, display statistical signatures of non-equilibrium complex systems, and whether such signatures, where present, are reproducible across signal types, seismic phases, and the methodological choices involved in extracting them. To this end, each recording was segmented into temporal windows using the automatic phase-picking AR-AIC method, and four coda onset definitions, and analysed through moment scaling, with the robustness of the resulting scaling exponents assessed against the choice of coda onset thresholds, the τ sub-interval used in the fit, and the applied filter band.

The evidence for anomalous diffusion in the moment scaling spectra ζ(q) is nuanced. Rather than a single, unconditional answer, this work finds that the reliability of anomalous scaling claims depends on the seismic phase, and, in several cases, the coda onset definition. The P-wave window yields the most consistent scaling exponents of the three windows; however, it is also the least robust to methodological choices unrelated to coda onset, with both the τ sub-interval sensitivity and the filter band sensitivity indicating that these exponents are better read as evidence of a genuine departure from normal diffusion than as precisely constrained physical quantities. The S-wave window shows negative but poorly constrained scaling exponents, and is comparatively robust to both the coda onset definition and the filter band. The coda window shows the weakest and most method dependent results, with scaling exponents that frequently change sign across coda onset definitions, particularly for displacement. This conditionality is one of the central findings of this work: claims of anomalous scaling in seismic ground motion cannot be treated as method-independent, and the systematic comparison across coda definitions developed here provides a framework for identifying which results are robust to these choices and which are not. To make the distinction between genuine physical signal and methodological artefact explicit, Table 11 summarises, for each seismic phase and signal type, which aspects of the estimated scaling exponents are considered robust and which are more likely to reflect segmentation, thresholding, or filtering choices rather than an intrinsic property of the ground motion signal. It should be emphasised that these findings pertain specifically to the L’Aquila event analysed here; given the reliance on a single earthquake, they should be regarded as a case study illustrating the proposed robustness framework rather than as evidence of universal scaling behaviour in seismic ground motion.

The observed scaling behaviour is compatible with interpretations of seismicity as a driven dissipative system far from equilibrium, including self-organized criticality and related statistical-physics approaches to seismicity [5,7]. However, the present study does not identify critical exponents, universality classes, or other signatures required to establish the existence of phase transitions, and therefore no such claim is made.

The most significant limitations of the present work are the dependence of the results on the choice of coda onset method and the inherent ambiguity in AR-AIC residuals between picking error and crustal velocity model inaccuracy. The analysis is further limited to a single seismic event, which prevents any assessment of how these findings generalise across different magnitudes and network geometries. Although the ITACA metadata for the L’Aquila dataset include manually reviewed phase picks, automatic pickers were deliberately adopted here to ensure methodological transferability to datasets lacking such annotations; a comparison with manual picks would nonetheless provide a useful upper bound on the achievable segmentation quality and represents a natural direction for subsequent investigation.

Future work could extend the present analysis to additional seismic events spanning a wider range of magnitudes and network geometries, enabling a systematic assessment of how scaling robustness depends on event size and station coverage. More broadly, applying the robustness framework developed in this work to a larger and more diverse set of events would clarify whether the stable configurations identified here correspond to genuinely generalisable physical signatures of anomalous diffusion in seismic ground motion, or remain specific to the case analysed.

From a wider perspective, seismological data also provide a valuable testbed for model reduction approaches grounded in response theory [31,32]. These methods enable the effective description of slowly evolving observables (capturing, e.g., the low-frequency envelope decay of relaxation processes), whose characteristic timescales are clearly separated from those of rapidly fluctuating fast variables (such as high-frequency signal oscillations associated with wave propagation and local scattering processes). This scale separation provides the theoretical guideline for reducing the complexity of the mathematical description of seismic events while retaining the essential information encoded in the underlying dynamics.

## Figures and Tables

**Table 1:** Parameters used for the ar_pick algorithm.

Parameter	Value	Description
lta_p	1.0 s	Long-term average window, P-wave
sta_p	0.1 s	Short-term average window, P-wave
lta_s	4.0 s	Long-term average window, S-wave
sta_s	1.0 s	Short-term average window, S-wave
m_p	2	AR model order, P-wave
m_s	8	AR model order, S-wave
l_p	0.1 s	Smoothing window length, P-wave
l_s	0.2 s	Smoothing window length, S-wave

**Table 2:** Moment scaling results for **acceleration** signals.

Window	Method	N	$\tau_{\max}$ (s)	ζ(1) ($R^{2}$)	ζ(2) ($R^{2}$)
P-wave ^∗^	–	117	2.27	1.28 (0.87)	2.00 (0.70)
S-wave	Rautian	180	4.92	−0.12 (0.27)	−0.27 (0.21)
	Arias	180	4.86	−0.13 (0.28)	−0.33 (0.29)
	Envelope	169	2.77	−0.13 (0.19)	−0.35 (0.22)
	Median	180	4.88	−0.15 (0.32)	−0.36 (0.38)
Coda	Rautian	141	2.88	−0.17 (0.40)	−0.28 (0.27)
	Arias	183	2.31	−0.11 (0.31)	−0.20 (0.26)
	Envelope	184	2.08	−0.03 (0.01)	0.02 (0.00)
	Median	184	2.08	−0.10 (0.12)	−0.17 (0.05)

^∗^ P-wave results are identical across all four coda onset methods, as the P-wave window boundaries depend only on the P- and S-wave onset times.

**Table 3:** Moment scaling results for **velocity** signals.

Window	Method	N	$\tau_{\max}$ (s)	ζ(1) ($R^{2}$)	ζ(2) ($R^{2}$)
P-wave ^∗^	–	120	2.08	2.34 (0.91)	4.55 (0.89)
S-wave	Rautian	180	4.92	−0.02 (0.02)	−0.17 (0.23)
	Arias	180	6.64	0.00 (0.00)	−0.14 (0.22)
	Envelope	150	2.16	0.10 (0.21)	−0.05 (0.01)
	Median	180	4.92	−0.02 (0.02)	−0.17 (0.23)
Coda	Rautian	137	3.13	−0.10 (0.21)	−0.29 (0.24)
	Arias	180	2.10	−0.15 (0.47)	−0.40 (0.45)
	Envelope	184	2.56	−0.01 (0.00)	−0.17 (0.12)
	Median	183	3.56	−0.12 (0.34)	−0.43 (0.32)

^∗^ P-wave results are identical across all four coda onset methods, as the P-wave window boundaries depend only on the P- and S-wave onset times.

**Table 4:** Moment scaling results for **displacement** signals.

Window	Method	N	$\tau_{\max}$ (s)	ζ(1) ($R^{2}$)	ζ(2) ($R^{2}$)
P-wave ^∗^	–	123	2.42	1.80 (0.96)	3.85 (0.95)
S-wave	Rautian	180	4.92	0.14 (0.47)	−0.24 (0.27)
	Arias	180	3.15	0.15 (0.47)	−0.25 (0.21)
	Envelope	168	2.21	0.22 (0.61)	−0.11 (0.07)
	Median	180	3.58	0.17 (0.57)	−0.18 (0.13)
Coda	Rautian	148	2.49	0.11 (0.43)	−0.25 (0.18)
	Arias	178	2.25	0.27 (0.93)	−0.09 (0.32)
	Envelope	182	2.04	0.34 (0.71)	0.46 (0.30)
	Median	185	3.04	0.25 (0.58)	0.32 (0.26)

^∗^ P-wave results are identical across all four coda onset methods, as the P-wave window boundaries depend only on the P- and S-wave onset times.

**Table 5:** Sensitivity of **acceleration** moment scaling exponents to the coda onset envelope threshold (baseline 30%), for the IT-2009-0009 event. For each affected coda method and alternative threshold value, z(1) and z(2) report the pointwise z-score (Equation (20)) at q=1 and q=2, and $z_{\max}$ the maximum z-score over the full range q∈[0.25,10.0], for the S-wave and coda windows.

		S-Wave			Coda		
Method	Value	z(1)	z(2)	$z_{\max}$	z(1)	z(2)	$z_{\max}$
Envelope	20%	0.47	0.47	0.55	0.94	1.77	2.06
	25%	0.22	0.50	1.15	0.80	0.77	0.85
	35%	0.56	0.47	0.56	0.18	0.22	0.75
	40%	0.10	0.28	0.72	1.23	1.40	1.93
Median	20%	0.65	0.75	1.56	0.18	0.15	1.03
	25%	0.61	0.75	0.75	0.06	0.31	0.79
	35%	0.24	0.25	0.39	0.13	0.43	0.74
	40%	0.24	0.25	0.39	0.41	0.04	0.66

**Table 6:** Sensitivity of **acceleration** moment scaling exponents to the coda end amplitude threshold (baseline 10%), for the IT-2009-0009 event, for the coda window. For each affected coda method and alternative threshold value, z(1) and z(2) report the pointwise z-score (Equation (20)) at q=1 and q=2, and $z_{\max}$ the maximum z-score over the full range q∈[0.25,10.0].

Method	Value	z(1)	z(2)	$z_{\max}$
Rautian	5%	0.00	0.00	0.06
	15%	0.01	0.00	0.12
	20%	0.47	0.30	0.59
Envelope	5%	0.01	0.00	0.11
	15%	0.80	1.22	1.46
	20%	0.94	1.15	1.86
Median	5%	0.03	0.00	0.14
	15%	0.11	0.25	0.43
	20%	0.24	0.13	1.03

**Table 7:** Sign test results for the P-wave τ sub-interval sensitivity analysis, testing the direction of ζ(q;upper)−ζ(q;lower) across all q∈[0.25,10.0] tested (N=40 per signal type).

Signal Type	*N*	Upper > Lower	Lower > Upper	*p*-Value
Acceleration	40	35	5	$6.9\times 10^{-7}$
Velocity	40	40	0	$9.1\times 10^{-13}$
Displacement	40	8	32	$9.1\times 10^{-5}$

**Table 8:** Sensitivity of **acceleration** moment scaling exponents to the filter band applied to the ground motion signal, for the **P-wave** window of the IT-2009-0009 event. For each alternative filter band configuration, z(1) and z(2) report the pointwise z-score (Equation (20)) at q=1 and q=2, and $z_{\max}$ the maximum z-score over the full range q∈[0.25,10.0]. All four coda onset methods yield identical results, given that P-wave window boundaries do not depend on the coda onset definition.

Filter Config	z(1)	z(2)	$z_{\max}$
near full-band configuration	2.99	4.27	4.88
narrow band (0.15–20 Hz)	1.15	2.69	5.24
wide band (0.02–50 Hz)	0.16	0.64	2.73

**Table 9:** Sensitivity of **acceleration** moment scaling exponents to the filter band applied to the ground motion signal, for the **S-wave** window of the IT-2009-0009 event. For each coda onset method and alternative filter band configuration, z(1) and z(2) report the pointwise z-score (Equation (20)) at q=1 and q=2, and $z_{\max}$ the maximum z-score over the full range q∈[0.25,10.0].

Method	Filter Config	z(1)	z(2)	$z_{\max}$
Rautian	near full-band configuration	0.62	0.43	0.62
	narrow band (0.15–20 Hz)	0.33	0.13	0.68
	wide band (0.02–50 Hz)	0.06	0.03	0.22
Arias	near full-band configuration	0.76	0.95	1.01
	narrow band (0.15–20 Hz)	0.56	0.61	1.15
	wide band (0.02–50 Hz)	0.87	0.90	0.91
Envelope	near full-band configuration	0.88	0.47	0.94
	narrow band (0.15–20 Hz)	0.64	0.28	0.65
	wide band (0.02–50 Hz)	0.63	0.60	0.68
Median	near full-band configuration	0.44	0.65	0.90
	narrow band (0.15–20 Hz)	0.48	0.76	0.78
	wide band (0.02–50 Hz)	0.77	0.88	0.99

**Table 10:** Sensitivity of **acceleration** moment scaling exponents to the filter band applied to the ground motion signal, for the **Coda** window of the IT-2009-0009 event. For each coda onset method and alternative filter band configuration, z(1) and z(2) report the pointwise z-score (Equation (20)) at q=1 and q=2, and $z_{\max}$ the maximum z-score over the full range q∈[0.25,10.0].

Method	Filter Config	z(1)	z(2)	$z_{\max}$
Rautian	near full-band configuration	0.10	0.30	1.39
	narrow band (0.15–20 Hz)	0.66	0.18	2.36
	wide band (0.02–50 Hz)	0.69	0.33	1.85
Arias	near full-band configuration	0.11	0.18	1.33
	narrow band (0.15–20 Hz)	0.30	0.27	1.69
	wide band (0.02–50 Hz)	0.14	0.02	1.54
Envelope	near full-band configuration	0.13	0.29	0.39
	narrow band (0.15–20 Hz)	0.80	1.08	1.97
	wide band (0.02–50 Hz)	0.01	0.14	0.75
Median	near full-band configuration	0.96	0.94	0.99
	narrow band (0.15–20 Hz)	0.58	0.74	0.96
	wide band (0.02–50 Hz)	0.22	0.21	0.26

**Table 11:** Summary of the reliability of the moment scaling exponents ζ(q) across seismic phases and signal types, based on the robustness framework of Section 2.4. The coda threshold and filter band sensitivity analyses were conducted on acceleration only; for velocity and displacement, robustness with respect to these two axes is not directly tested and is not assumed.

	Acceleration	Velocity	Displacement
P-wave	Positive, growing ζ(q) plausibly reflects the impulsive P-wave transient; magnitude at high *q* unreliable (τ sub-interval, filter band both fragile)	Same qualitative signature as acceleration; least reliable of the three signal types (strongest τ sub-interval instability)	Same qualitative signature as acceleration; direction of τ sub-interval deviation reverses at q≈1.25, high-*q* values unreliable
S-wave	Weak, near-zero ζ(q) is a robust, likely genuine feature (stable across coda method and filter band)	Weak, near-zero ζ(q); robust across coda method	Not reliable without specifying the method
Coda	Modest ζ(q), mostly stable across coda method and filter band; one exception (Rautian, narrow band)	Modest, negative ζ(q) across coda methods	Sign of ζ(2) depends on coda method: not reliable without specifying the method

## Data Availability

The data presented in this study are available in the Italian Accelerometric Archive (ITACA) at https://itaca.mi.ingv.it (accessed on 1 March 2026), reference event IT-2009-0009 (L’Aquila, $M_{w}\;6.1$, 6 April 2009). The analysis code is available at https://github.com/giulianaparadiso99/tesi-seismic-analysis.

## Appendix A. Phase Picking and Signal Segmentation Results

The following tables report aggregate statistics for AR-AIC phase picking, adaptive search windows, and quality control, computed across the entire network (up to 186 three-component records from 62 stations).

**Table A1:** Hypocentral distance bins and half-widths of the adaptive AR-AIC search windows.

Bin	Range (km)	N Stations	$\Delta t_{P}$/$\Delta t_{S}$ (s)
Bin 1 (near)	(0, 82.62]	21	±3/±6
Bin 2 (regional)	(82.62, 175.66]	20	±5/±10
Bin 3 (far)	(175.66,∞)	21	±7/±14

**Table A2:** Mean residuals between onset times detected by AR-AIC and theoretical arrival times from CRUST1.0, for the three signal types.

Signal	Phase	Mean ± Std (s)	Min (s)	Max (s)
Acceleration	P-wave	0.55±1.93	−6.09	2.21
	S-wave	3.64±5.36	−14.00	12.87
Velocity	P-wave	0.23±1.92	−5.16	3.86
	S-wave	3.51±5.83	−14.00	12.35
Displacement	P-wave	−0.61±2.32	−6.22	4.96
	S-wave	2.09±7.04	−14.00	13.56

**Table A3:** Number of components failing each quality control check (AR-AIC, no-threshold configuration), for the three signal types and the four coda onset detection methods. MonoP, MonoS, and SNR P are independent of the coda method and are shown once per signal type. Denominators differ across methods due to missing windows.

Signal	Method	Peak	Mono P	Mono S	SNR P	SNR S
Acceleration	Rautian	27/180 (15.0%)	159/186 (85.5%)	159/186 (85.5%)	0/117 (0.0%)	0/126 (0.0%)
	Arias	15/180 (8.3%)				0/126 (0.0%)
	Envelope	49/170 (28.8%)				0/121 (0.0%)
	Median	21/180 (11.7%)				0/126 (0.0%)
Velocity	Rautian	32/180 (17.8%)	153/186 (82.3%)	168/186 (90.3%)	8/120 (6.7%)	0/126 (0.0%)
	Arias	12/180 (6.7%)				0/126 (0.0%)
	Envelope	53/151 (35.1%)				0/111 (0.0%)
	Median	26/180 (14.4%)				0/126 (0.0%)
Displacement	Rautian	42/180 (23.3%)	162/186 (87.1%)	162/186 (87.1%)	31/117 (26.5%)	3/120 (2.5%)
	Arias	20/180 (11.1%)				3/120 (2.5%)
	Envelope	81/168 (48.2%)				3/117 (2.6%)
	Median	34/180 (18.9%)				3/120 (2.5%)
